# Targeting Protein Kinase C Downstream of Growth Factor and Adhesion Signalling

**DOI:** 10.3390/cancers7030836

**Published:** 2015-07-15

**Authors:** Catríona M. Dowling, Patrick A. Kiely

**Affiliations:** 1 Department of Life Sciences, Materials and Surface Science Institute and Stokes Institute, University of Limerick, Limerick 78666, Ireland; 2 Health Research Institute (HRI), University of Limerick, Limerick 78666, Ireland

**Keywords:** growth factor signaling, receptor tyrosine kinase, G-protein coupled receptors, integrins, Protein Kinase C, cell proliferation, cell adhesion.

## Abstract

The signaling outputs of Receptor Tyrosine Kinases, G-protein coupled receptors and integrins converge to mediate key cell process such as cell adhesion, cell migration, cell invasion and cell proliferation. Once activated by their ligands, these cell surface proteins recruit and direct a diverse range of proteins to disseminate the appropriate response downstream of the specific environmental cues. One of the key groups of proteins required to regulate these activities is the family of serine/threonine intracellular kinases called Protein Kinase Cs. The activity and subcellular location of PKCs are mediated by a series of tightly regulated events and is dependent on several posttranslational modifications and the availability of second messengers. Protein Kinase Cs exhibit both pro- and anti-tumorigenic effects making them an interesting target for anti-cancer treatment.

## 1. Introduction

The composition and organization of the extra-cellular matrix (ECM) regulates cell behaviour and tissue morphogenesis. It does this by regulating integrin clustering and also by controlling the availability of bioactives such as growth factors which control critical parameters such as cell migration, cell differentiation, cell polarity and cell proliferation [1,2]. The components of the ECM are regulated by hundreds of genes in the cells of the tissue and stroma [3]. Tumour cells respond to subtle changes in the composition of the ECM but can also influence the composition of the ECM by mediating dysregulation of particular sets of genes. This confers advantages to the tumour cells influencing changes in cytoskeletal dynamics, cortical tension, and microtubule turnover and enhances the transformed phenotype and promotes cancer progression [4]. How the cells detect and respond to changes in the ECM is mediated by a series of cell surface receptors that continuously sample the external environment. Signals from these cell surface receptors converge to recruit the cells adhesion and migratory machinery [5,6,7].

Receptor tyrosine kinases (RTKs) form a major part of the enzyme-linked family of receptors and include receptors for epidermal growth factor (EGF), fibroblast growth factor (FGF), platelet derived growth factor (PDGF), vascular endothelial growth factor (VEGF), hepatocyte growth factor (Met), glial cell line-derived neurotrophic factor (Ret), neurotrophins (Trks) and the insulin receptor family (IR) which includes the type I insulin-like growth factor receptor (IGF-1R) (reviewed in [5,8,9]). The activity of RTKs is under tight control by several modes of regulation such as transcriptional and post-transcriptional regulation and receptor internalisation. Once activated by their ligands, RTKs initiate diverse downstream signal transduction events to promote cell migration, but also proliferation, differentiation and the regulation of cell metabolism (reviewed in [5,8,10]).

Adhesion to the ECM is mediated by cell surface receptors such as integrins, members of the immunoglobulin superfamily and tyrosine kinase receptors [2]. Integrins are αβ heterodimeric receptors that are composed of a large extracellular domain that binds ECM components and a short cytoplasmic tail that links to the actin cytoskeleton [11]. There have been 18 α subunits and 8 β subunits identified that associate with each other to generate 24 different receptors with distinct ligand specificities. For example, fibronectin which possesses both leucine-aspartic acid-valine (LDV) and arginine-glycine-aspartic acid (RGD) motifs can bind to many integrins (all five αV integrins, α5β1, α8β1, α4β1, α4β7 and α9β1), laminin binds α1β1, α2β1, α3β1, α6β1, α7β1, α10β1, α11β1 and α6β4 and collagen binds α1β1, α2β1, α10β1 and α11β1 [12]. Integrins are the site of focal adhesion formation and these structures are required for polarised cell migration characterised by asymmetric adhesion dynamics with formation of adhesions at the leading edge and disassembly of adhesions at rear of the cell [13]. Like RTKs, integrins can independently propagate intracellular signals but cooperate with RTKs to initiate a cellular response through downstream signalling pathways to promote adhesion and migration.

There are many ways by which these receptor groups cooperate. Central to these events is the requirement for the integration of multiple signalling pathways. Clustering of integrins and phosphorylation of RTKs on specific tyrosine residues in the cytoplasmic domain creates binding sites for intracellular signalling molecules and facilitates the recruitment of adaptor proteins and specialized protein docking modules, including those with SH2 domains, plextrin homology (PH) domains, PDZ domains, and C2 domains (PKCs) [14]. Adaptor proteins in particular are strategically positioned at key steps in signalling pathways and function to disseminate and amplify signals accurately downstream of growth factor and adhesion receptors. Adaptor proteins generally do not possess enzymatic activity but facilitate the creation of signalling complexes by bringing protein-binding partners together. The scaffolding of specific proteins in close proximity facilitates the reciprocal modulation of protein function and subsequent regulation of signalling events so that an appropriate response can be elicited.

An important function of activated cell surface receptors is the recruitment of intracellular kinases [5,15]. This recruitment leads to a cascade of downstream signalling and has a major influence on cell characteristics such as cell adhesion, proliferation, migration and invasion [16,17]. Focal adhesions are the converging point for growth factor and adhesion receptor signaling [18,19,20]. These are large dynamic macromolecular assemblies with signalling components and mechanical components and focal adhesions are assembled very precisely after the clustering of integrins on the cell surface. Integrin clustering is sufficient to promote the phosphorylation of focal adhesion kinase (FAK) on Tyr397. This facilitates the binding of the Src homology 2 (SH2) domains of Src family protein tyrosine kinases (Src-family PTKs) to promote the phosphorylation of FAK at secondary sites ensuring full activation of FAK. Once activated, FAK interacts directly with other non-receptor tyrosine kinases, cell surface receptors, cytoskeletal proteins and other adaptor proteins. We and others have characterised the interaction between Beta1 integrins and the IGF-IR and have shown that a WD repeat containing the scaffolding protein RACK1 mediates crosstalk between the IGF-IR and adhesion receptors by orchestrating the recruitment of a series of proteins to regulate focal adhesions [21,22,23,24,25,26,27,28].

A key subgroup of these intracellular kinases are Protein Kinase Cs (PKC), a family of serine/threonine kinases which play key roles in several signalling pathways [29,30,31,32]. This group of proteins are expressed in many different tissue types and hence have a diverse range of biological functions [33,34]. At focal adhesions, PKCs have been shown to interact with several structural, mechanical and regulatory proteins that are central to the establishment, maintenance and disassembly of focal adhesions (reviewed in [35]).

## 2. Protein Kinase C: Structure, Function and Activity

Three different subfamilies of the PKCs exist; the classical /conventional PKC isozymes (cPKC) α, βI, βII and ϒ, the novel PKC isozymes (nPKC)δ, ε, θ, η and μ and the atypical PKC isozymes (aPKC) ι(human)/λ(mouse) and ζ [29,36]. The most extensively studied and consequently most understood are the conventional PKCs [31,36,37], the major focus of this review. PKCs are ubiquitously expressed but there are differences in expression levels in different tissues. Once activated the distribution, activity and cellular location of PKCs is cell dependent and the process is tightly regulated by several signalling pathways. Scaffolding proteins play an important role in facilitating PKC activity by converging different signalling cascades and by orchestrating specific protein-protein interactions [33]. The PKC isozymes are responsible for mediating several biological processes including cell-cycle regulation and cell survival [33,34,38]. PKCs also play a critical role in mediating cell attachment, cell adhesion and cell spreading by regulating integrin signalling pathways [35,39,40,41]. During this process, several of the PKC isozymes are recruited into developing focal adhesions and form central components of the integrin-signalling pathway [35,42,43,44].

**Figure 1:**
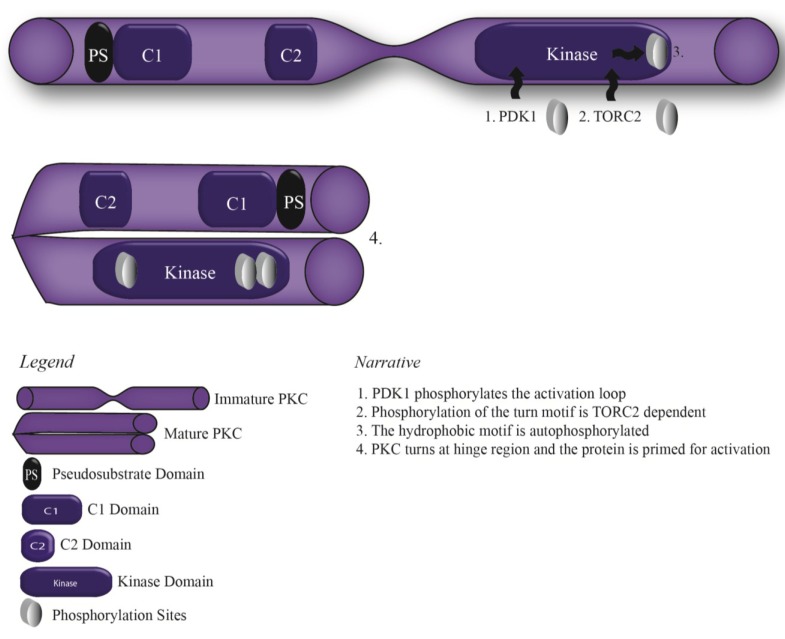
Priming of Protein Kinase C in to its inactive mature conformation.

All members of the PKC family share common basic structures; a flexible hinge segment linking a cell membrane targeting N-terminal regulatory domain to a C-terminal catalytic domain [45,46]. The regulatory domain maintains the enzyme in an inactive conformation with two discrete membrane targeting modules, termed C1 and C2 [47]. The C1 domain binds diaclyglycerol (DAG) in all but the atypical isozymes and the C2 domain binds anionic lipids with the conventional isozymes also binding phosphatidylserine (PS) and calcium [48,49,50,51,52]. The maturation of cPKCs into this inactive conformation is dependent on phosphorylation steps at three highly conserved sites termed: the activation loop, the turn motif, and the hydrophobic motif [53,54]. Phosphoinositide-dependent kinase-1 (PDK1) phosphorylates newly synthesised cPKC on a threonine residue at the activation loop which positions the active site for catalysis [47,55,56]. This phosphorylation triggers phosphorylation of the turn motif and consequently autophosphorylation of the hydrophobic motif, leading to the optimal stability of the enzyme [57,58]. The maturation of cPKC primes it for activation allowing changes in intracellular calcium levels to recruit the C2 domain to the membrane followed by the binding of DAG to the C1 domain and so the release of the active site [47] (Figure 1).

## 3. Activation of PKCs: Receptor Tyrosine Kinases, G-protein Coupled Receptors and Integrins

The mechanisms underlying PKC activation have been well studied and are triggered by changes the in intracellular cofactors of PKCs downstream of signals that stimulate RTKs, G-protein-coupled receptors (GPCR) and integrins [59].

**Figure 2:**
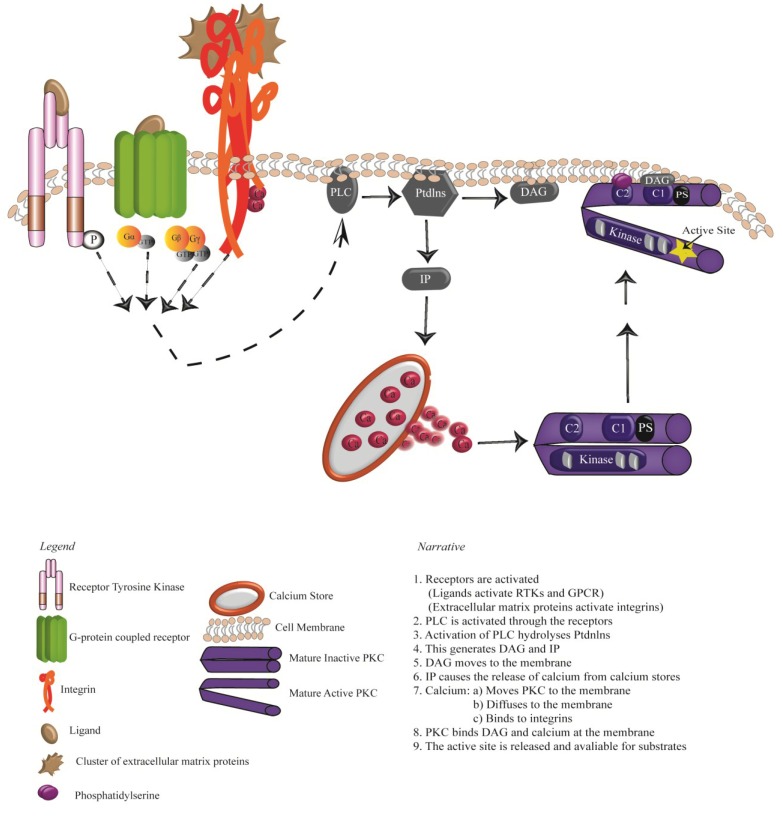
Activation of mature Protein Kinase C.

There are 58 known human RTKs which fall into 20 subfamilies, many of which are believed to play a role in the activation of PKCs [8,9]. Growth factors were thought to activate various RTKs through dimerization but the intracellular signalling responses of RTKs were largely unknown. In 1984, EGF was found to stimulate the RAS oncoprotein [60] and shortly after, phospholipase c (PLC) was discovered as the first substrate that directly interacts with activated EGFR [61]. Dimerization of RTKs by growth factors causes the auto phosphorylation of their cytoplasmic tyrosine domains, creating a series of high affinity docking sites for signalling proteins such as phosphoinositide 3-kinase (PI3-kinase) and PLC [62,63]. PI3-Kinases are a large family of kinases consisting of three classes and multiple subunits [64]. They contain a regulatory and catalytic subunit; the p85 regulatory subunit either binds directly with activated RTKs through its SH2 domain or binding may be mediated through phosphoproteins such as the insulin receptor substrate [63,65]. PI3-kinase phosphorylates the 3 position hydroxyl group of the inositol ring of phosphatidylinositols (PtdIns) [66]. PLC isozymes are divided into three classes, β, γ and δ, differing on their mechanisms of activation. PLCβ (β1 and β2) is activated by G-proteins, little is known about the activation of δ isoymes (δ1 and δ2) and PLCγ (γ1 and γ2) is activated by RTKs [67]. PLCγ translocates from the cytosol to the membrane, where its N-terminal and C-terminal SH2 domain binds to the phosphorylated tyrosine site of the RTK [68]. Upon activation, PLCγ hydrolyse Ptdlns generating two secondary messengers, namely, DAG and inositol phosphates (IPs). IPs stimulate the release of calcium from intracellular stores, activating cPKCs and translocation of the protein to the cell membrane at which the C2 domain can bind to PS, followed by the binding of DAG to the C1 domain (Figure 2) [69,70]. The binding of the C2 domain to PS in novel PKCs does not require calcium and thus their membrane recruitment and activation is calcium- independent [46]. This process of PKC activation has been well established for many growth factors such as EGF, PDGF and nerve growth factor (NGF) [59,70,71]. Following stimulation of PDGF receptors, PLCγ can bind to the receptor at tyrosine residue Y1021 and PI3-kinase can bind at tyrosine Y740 and Y751, resulting in the translocation of PKCε [72,73]. PKCλ can also translocate from the nucleus to the cytosol when activated through growth factors PDGF and EGF but this translocation only involves PI3-kinase signalling [74]. EGF treatment of cells has also shown to translocate PKCα and PKCγ to the membrane with no effect on the translocation of PKCζ [75,76]. Both EGF and NGF activation of PKCε in neuronal cells provides a positive signal for neurite outgrowth [75,77].

The role of PKCs in regulating growth factor signalling has been well documented. For example, activation of PKCs via EGF results in the direct phosphorylation of the EGFR at Thr 654 leading to a decrease in ligand affinity and receptor activity [78,79,80,81]. It is believed that PKCs regulate whether the cellular response to EGF is pro-mitogenic or pro-motility [82,83,84]. PKC isozymes are also known to regulate the HGF receptor c-Met. PKCα controls the trafficking of c-Met to perinuclear compartments in a microtubule dependent manor [85] and PKCε is required for the c-Met activation of ERK [86]. Activation of ERK downstream of PKCε and their localization to focal adhesions is required for PKCε induced adhesion and migration [87]. In contrast, it has also been shown that PKCδ and PKCε can inhibit tyrosine phosphorylation of c-Met by phosphorylating c-Met at Thr 985 [59]. It is thought that these isozymes may be functioning to regulate c-Met receptor signalling as PKCδ and PKCε mediated phosphorylation at Thr 985 is promoted by HGF itself and rapidly removed by protein phosphatase 2 (PP2A) [88].

G-protein-coupled receptors have a characteristic core composed of seven transmembrane α helices weaving in and out of the membrane and with over 800 members they represent the largest family of cell-surface molecules involved in signal transmission [89,90]. Many different agonists stimulate GPCRs and upon stimulation of the extracellular side of the receptor introduce a conformational change allowing for the interaction of the heterotrimeric G-proteins with the intracellular sites on the receptor [91]. G-proteins contain three subunits; α,β and γ, when bound to the receptor guanosine diphosphate (GDP) association with the Gα subunit is replaced with guanosine triphosphate (GTP), which in turn leads to the dissociation of Gα from the Gβγ subunit and hence activation of the protein [92]. At present there are 17 known Gα, 5 β and 12 γ subunits with the Gα subunits divided into four major families Gα_s_, Gα_i_, Gα_q_ and Gα_12/13_ [92,93]. The Gα_q_ family play a major role in the activation of PKCs through the stimulation of PLC-β hydrolysis of phosphatidylinositol biphosphate (PIP_2_) producing inositol triphosphate (IP_3_) and DAG, the two major secondary messengers required for PKC recruitment and activation (Figure 2) [89]. Traditionally it was thought that the Gβγ complex solely served to bind the Gα subunit to prevent spontaneous signalling, however, in more recent years it has emerged as an activator of the PLC signalling pathway and hence, PKC activation (Figure 2) [94].

Protein Kinase C appears to be a key intermediate in integrin mediated signalling and while much of the research on integrins and PKCs has focused on the regulation of integrin activity by PKCs, recent studies also highlight that integrins play a central role in mediating activation of PKCs [95,96]. Extracellular matrix proteins cluster and activate integrins which in turn induce the PLC signalling cascade and the activation of PKCs, in a process termed outside-in signalling (Figure 2). This activation of PKCs results in a mobilization of calcium levels within the cell and consequently further activation of integrins, a phenomenon referred to as inside-out signalling [95,97,98,99]. Consequently, PKC activation has a central role to play in the establishment and maintenance of focal adhesions downstream of clustered integrins (reviewed in [35]). Inhibition of PKC activity in many cell types results in a reduction in cell spreading, a consequence of reduced focal adhesion formation in the cells [44,100].

## 4. Oncogenic Signalling Downstream of RTKS, GPCRs and Integrins

Research into PKC signalling intensified when it was discovered that PKC is a high-affinity intracellular receptor for phorbol-ester tumour promotors such as TPA [37,101,102]. This strongly suggested that PKC activation promoted tumorigenesis induced by carcinogens. PKC signalling and oncogenic signalling converge and contribute to the transformed phenotype [102,103,104], however it remains unclear as to whether tumour promotion is a result of changes in specific activity, or by changes in the expression of the protein [33,105,106,107,108]. Confusion also arises as immunohistochemical and biochemical studies indicate that altered expression of the PKC isozymes is variable and depends on the cancer cell type [32,42,59,109]. This could suggest that perhaps a change in both the activity and expression is associated with the transformed phenotype. In an attempt to delineate this confusion this review will discuss the most recent findings presented for each conventional isozyme.

When examining the expression levels of PKCα there are contradictory results emanating from different tissue types. Over expression of the isozyme is reported in tissue samples from prostate, endometrial, urinary bladder and hepatocellular cancers while down regulation has been observed in basal cell and colon cancer [110,111,112,113,114,115,116]. In breast cancer, up/down regulation of PKCα has been suggested to be dependent on the specific subtypes of the disease [117,118,119]. However, taken together, the majority of studies suggest PKCα plays a role in increasing the proliferation and invasive capacity of cancers and many PKCα inhibitors have shown to reverse the phenotype [120,121,122]. Studies propose PKCα facilitated invasion can occur in a number of different ways; through inhibition of protein complexes at cell junctions, inhibition and mobilisation of hemidesmosomes mediated through the β4 integrin, and PKCα can introduce changes in β1 integrin mediated cell matrix junctions [121,123,124]. More specifically in breast cancer, it has been proposed that alteration in the subcellular localization of PKCα results in a change in the desmosomal adhesive state of the cells potentially leading to a loss in cell–cell adhesion and a transition from a normal to a malignant phenotype [125].

Two splice variants have been described for PKCβ and again the expression levels of both variants differ between tissue types. Loss of PKCβ is observed in malignant melanocytes and melanoma cell lines [126]. The expression of PKC β1 and β2 in breast, gastric and colon cancer has been subject to much debate and there are many studies presenting arguments for both up and down regulation of the isozyme in the diseases [127,128,129,130,131,132]. Despite these conflicting studies, the role of PKCβ in angiogenesis has been well documented. The isozyme plays a role in mediating VEGF signalling and its inhibition in this pathway results in decreased endothelial cell proliferation and reduction of neovascularization in malignant tumours [133,134].

PKCγ is predominantly expressed in neuronal tissues and there is very little evidence to suggest a role for PKCγ in tumorigenesis. In certain forms of B-cell lymphomas, PKCγ expression has proven to be a positive prognostic factor [135]. Mammary epithelial cells overexpressing PKCγ acquire a malignant phenotype *in vivo* [136], however, its role in breast cancer formation has not yet been established. More research into the role of PKCγ in tumour formation needs to be conducted in order to establish what role, if any, it plays in the development of tumours.

## 5. Consequences of Targeting PKC

**Figure 3:**
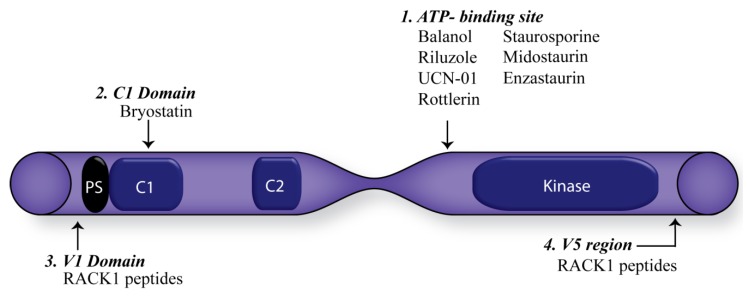
Therapeutic approaches targeting Protein Kinase C.

There have been many efforts made in targeting PKCs for anti-cancer treatments [33,137]. A number of different approaches have been explored in an attempt to create selective modulators for the PKC isozymes including; ATP competitive small molecule inhibitors, phorbol esters and derivative activators and inhibitors which mimic the binding of diacylglycerol and peptides that disrupt protein-protein interactions between PKC and its corresponding RACK (Figure 3) [33,132,137,138].

The first ATP-binding site inhibitor, staurosporine, was developed over 30 years ago. This compound binds to all PKC isozymes but also binds non-specifically to several other serine/threonine kinases [139]. Midostaurin (PKC412), a derivative of staurosporine, was subsequently developed in an attempt to design a more isozyme specific ATP-binding site inhibitor. The compound exhibits an increased specificity for the conventional and atypical PKCs but also inhibits other tyrosine kinase pathways [140]. In preclinical trials reports suggest that PKC412 may act as a radio-sensitizer for human xenografts through the blockade of the P13K/Akt pathways [141]. Enzastaurin, emerged as another ATP-binding compound, thought to be specific to PKCβ. While demonstrating a 20-fold more powerful inhibiton of PKCβ, it is now believed that it can also inhibit other PKC isozymes [142]. Enzastaurin prevents angiogenesis through alteration of the VEGFR signalling cascade [143] and is currently in clinical trials for brain malignancies [144].

The C1 domain of the regulatory region of PKC’s has also been targeted. A number of compounds have been developed over the years but the most famous of these compounds, bryostatin, a naturally occurring macrolactone, mimics the binding of DAG and has pan PKC activity [145]. Preclinical trials looked promising with bryostatin showing an effect on tumours including melanoma, leukemia, lymphoma and lung cancer [146]. Despite this, the drug has proven to be disappointing in clinical trials, as it showed very little efficacy in reducing tumour growth, even when combining the drug with other cytotoxic drugs and has since been suspended [37].

The translocation of PKCs to their subcellular locations is an imperative part of PKC activity, leading to the development of modulators that target the interaction sites of these shuttle proteins. The C2 domain is the main focus for designing inhibitors against protein-protein interactions; this is owed to the founding work of the Mochley-Rosen lab, who demonstrated that unique sequences within the C2 domain are a “hot-spot” for several protein-protein interactions [147]. However, other protein-protein interactions have been delineated in regions between the C1, C2, C3, and C4 regions, the V2, V3 and V5 regions as well as between the C1a and C1b subdomains [33]. These protein-protein interactions are the subject of a new generation of PKC targeting. For example, a peptide derived from the V5 region, which inhibits the interaction of PKCβII and RACK1 has shown to prevent cardiac dysfunction and death in rat models of post heart failure and inhibit neoangiogenesis in a xenograft mouse model of prostate cancer [148,149]. 

## 6. Conclusions

The role RTKs, GPCRs and integrins play in activating PKCs is well characterised and is a key feature of cell proliferation and oncogenic signalling. Traditionally, research into the role of different PKC isozymes in cancer was primarily based on the assumption that increased PKC activation and expression promotes carcinogen induced tumorigenesis [103,150,151,152,153].

However, increasing evidence suggests that many PKC isozymes can act as both tumour suppressors and oncogenes. For example, PKCδ has pro-apoptotic effects leading to the belief that it is acting as a tumour suppressor but it has also been linked to the progression of pancreatic and lung cancers [154,155,156]. Similarly, PKCζ overexpression in colon cancer cell lines decreases tumour formation in nude mice while loss of PKCζ is also associated with decreased tumorigenicity [157,158]. Many efforts have been made to create molecules that target PKCs for cancer therapy, but attempts to date have been largely unsuccessful [159]. This may owe to the difficulty in creating inhibitors that target specific PKC isozymes in cancer, coupled with the challenges associated with indirectly disrupting the physiological role of PKCs in normal cells. PKC mutations exist in a diverse range of cancers, existing in the entire coding region with no hotspots. However, it is worth noting that recent pioneering work conducted in the Newton lab surprisingly demonstrated the majority of mutations found in PKCs resulted in a loss of function and none were activating [160]. Further to this, meta-analysis on patients with non-small cell lung cancer in controlled trials of PKC inhibitors, combined with chemotherapy in comparison with chemotherapy alone, revealed that PKC inhibitors significantly decreased response rates and disease control rates [161].

The ideas presented in this review highlight the challenges in targeting PKCs; clearly defining which PKC isozyme is displaying a tumour suppressor and/or tumour promotor roles and in which tissues proves to be a difficult task. Protein Kinase Cs are key intracellular targets for growth factor and adhesion signaling pathways so perhaps a refocus and revisit of the pathways upstream of PKCs presents a more favourable approach to targeting this group of kinases.
